# Cost Effectiveness of Watch and Wait Versus Resection in Rectal Cancer Patients with Complete Clinical Response to Neoadjuvant Chemoradiation

**DOI:** 10.1245/s10434-021-10576-z

**Published:** 2021-09-16

**Authors:** Christina Liu Cui, William Yu Luo, Bard Clifford Cosman, Samuel Eisenstein, Daniel Simpson, Sonia Ramamoorthy, James Murphy, Nicole Lopez

**Affiliations:** 1 School of Medicine, University of California, San Diego, La Jolla, CA USA; 2 Department of Surgery, Division of Colon and Rectal Surgery, University of California, San Diego Health Systems, La Jolla, CA 92093-0987 USA; 3 Veterans Affairs San Diego Medical Center, San Diego, CA USA; 4 Department of Radiation Medicine and Applied Science, University of California, San Diego, La Jolla, CA USA

## Abstract

**Background:**

Watch and wait (WW) protocols have gained increasing popularity for patients diagnosed with locally advanced rectal cancer and presumed complete clinical response after neoadjuvant chemoradiation. While studies have demonstrated comparable survival and recurrence rates between WW and radical surgery, the decision to undergo surgery has significant effects on patient quality of life. We sought to conduct a cost-effectiveness analysis comparing WW with abdominoperineal resection (APR) and low anterior resection (LAR) among patients with stage II/III rectal cancer.

**Methods:**

In this comparative-effectiveness study, we built Markov microsimulation models to simulate disease progression, death, costs, and quality-adjusted life-years (QALYs) for WW or APR/LAR. We assessed cost effectiveness using the incremental cost-effectiveness ratio (ICER), with ICERs under $100,000/QALY considered cost effective. Probabilities of disease progression, death, and health utilities were extracted from published, peer-reviewed literature. We assessed costs from the payer perspective.

**Results:**

WW dominated both LAR and APR at a willingness to pay (WTP) threshold of $100,000. Our model was most sensitive to rates of distant recurrence and regrowth after WW. Probabilistic sensitivity analysis demonstrated that WW was the dominant strategy over both APR and LAR over 100% of iterations across a range of WTP thresholds from $0–250,000.

**Conclusions:**

Our study suggests WW could reduce overall costs and increase effectiveness compared with either LAR or APR. Additional clinical research is needed to confirm the clinical efficacy and cost effectiveness of WW compared with surgery in rectal cancer.

Over 700,000 cases of colorectal cancer are diagnosed annually worldwide, with 30% of tumors arising in the rectum.^1,2^ One-third of rectal cancer patients present with locally advanced disease.^3^ Current standard of care for locally advanced rectal cancer consists of neoadjuvant radiation, with or without chemotherapy, followed by radical resection.^4^ Extent of resection, low anterior resection (LAR) or abdominoperineal resection (APR), depends on tumor relation to the sphincter complex.

However, evidence suggests that some patients may be able to avoid surgery. Data from prospective clinical trials demonstrate that up to 25% of patients have a pathological complete response (pCR; no viable tumor on pathological examination) after neoadjuvant chemoradiation.^5–8^ These patients have demonstrated lower odds of local or distant recurrence and greater odds of 5-year disease-free survival.^5,9,10^ Given the significant decrease in quality of life associated with surgery, surgeons are increasingly considering whether patients with pCR may represent a cohort that could have avoided surgery.^11,12^ However, since pCR can only be determined after surgical resection, post-neoadjuvant selection criteria for patients typically relies on clinical complete response (cCR). While an imperfect approximation, cCR can act as a surrogate marker for pCR, and is determined using post-chemoradiation clinical, endoscopic, or radiographic evaluations. Watch and wait (WW) protocols for patients with cCR have gained increasing popularity since described by Habr-Gama et al. in 2004.^13^ Patients with cCR undergoing a WW protocol have approximately 10% risk of distant recurrence and 20% risk of local regrowth;^14,15^ the majority of patients with regrowth can undergo salvage surgery (95%).^14,16^ Similarly, when comparing all patients undergoing surgery with all patients managed with the WW strategy, WW patients demonstrate non-inferior local recurrence/regrowth-free survival.^17^ These data indicate that WW is safe and feasible for patients with cCR after neoadjuvant therapy. This is true even despite the heterogeneity of staging and surveillance strategies used in current data.^15,18^

WW protocols may also confer higher patient quality of life. Increased colostomy-free survival, as demonstrated by Renehan et al., is a perceived benefit among patients eligible for the WW treatment approach.^17^ This is also supported by a cross-sectional study by the Dutch Prospective Data Collection Initiative on Colorectal Cancer (PLCRC), in which WW was preferred by the majority of patients. Indeed, WW had one of the highest perceived health utility scores out of all interventions.^19^ In comparison, surgical intervention has well-described long-term negative impacts on patient quality of life, with patients undergoing LAR reporting an approximately 17% decrease in health utility during the first few months after surgery.^20^ Despite the fact that patient quality of life can be greatly impacted by surgery, helping patients consider strategies for optimizing quality of life in medical decision-making processes can often fall short.^21–23^

In this study, we aimed to compare quality of life (as measured in quality-adjusted life-years [QALYs]), efficacy, and cost of treatment among patients undergoing WW versus LAR or APR.

## Methods

### Cost-Effectiveness Model

**Fig. 1:**
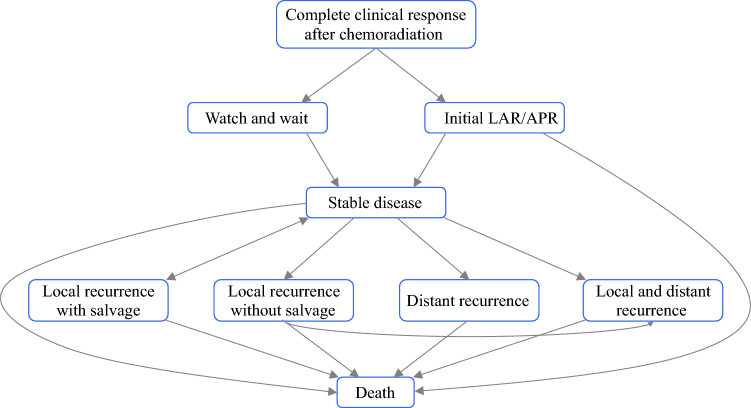
Transition state diagram summarizing the Markov simulation model employed in our study. *LAR* low anterior resection, *APR* abdominoperineal resection

We created Markov microsimulation models for patients with cCR after neoadjuvant chemoradiation for locally advanced rectal cancer. The models simulate the outcomes of 10,000 patients with stage II/III rectal cancer with a cCR after neoadjuvant chemoradiation opting for WW or radical surgery. Given the different quality of life associated with APR and LAR, we created separate models for each surgical option (WW versus APR, and WW versus LAR). The models included six distinct heath states: stable disease, local progression with salvage surgery, local progression without salvage surgery, distant progression, both local and distant progression, and death. Our local and distant progression health state allowed us to model concurrently diagnosed local and distant recurrence, local recurrence with subsequent finding of distant recurrence, and distant recurrence with subsequent findings of local recurrence. Each of these events had distinct transition probabilities associated with them (Table 1). Patients all started in the ‘stable disease’ state in either the WW or initial surgery arms and could stay in this state, transition to one of the progression states, or die (Fig. 1). Patients could die from cancer-related or non-cancer causes.

**Table 1:** Transition probability parameters used in the Markov model and their respective citations

Model parameter	WW			LAR			APR		
	Value	SD	Citation	Value	SD	Citation	Value	SD	Citation
*Probabilities (β Distribution)*									
Perioperative death	Reflects APR or LAR probabilities, depending on the model			0.035	0.007	Marijnen et al., 2002^47^	0.035	0.007	Marijnen et al., 2002^47^
Local recurrence/regrowth, 2 years	0.19	0.04	Dossa et al., 2017^14^	0.016	0.003	Dossa et al., 2017^14^	0.016	0.0032	Dossa et al., 2017^14^
Local recurrence/regrowth, 5 years	0.24	0.05	van der Valk et al., 2018^16^ Smith et al., 2019^15^	0.010	0.002	Miller et al., 2020^41^	0.010	0.002	Miller et al., 2020^41^
Salvage for local recurrence	0.94	0.19	Dossa et al., 2017^14^	0.590	0.12	Ikoma et al., 2017^53^	0.59	0.12	Ikoma et al., 2017^53^
Distant recurrence, 5-year	0.10	0.02	Dossa et al., 2017^14^	0.079	0.016	Dossa et al., 2017^14^	0.079	0.016	Dossa et al., 2017^14^
Concurrent diagnosis of distant recurrence if local recurrence	0.06	0.01	van der Valk et al., 2018^16^	0.056	0.011	van der Valk et al., 2018^16^	0.056	0.011	van der Valk et al., 2018^16^
Concurrent diagnosis of local recurrence if distant recurrence	0.17	0.03	van der Valk et al., 2018^16^, ^26^	0.17	0.034	van der Valk et al., 2018^16^	0.17	0.034	van der Valk et al., 2018^16^
Distant recurrence following local recurrence, 3-year	0.11	0.02	van der Valk et al., 2018^16^	0.11	0.023	van der Valk et al., 2018^16^	0.11	0.023	van der Valk et al., 2018^16^
Mortality after local recurrence with salvage, 5-year	0.50	0.10	Rao et al., 2017^43^	0.50	0.10	Rao et al., 2017^43^	0.50	0.10	Rao et al., 2017^43^
Mortality after local recurrence without salvage, 5-year	0.70	0.14	Rao et al., 2017^43^	0.70	0.14	Rao et al., 2017^43^	0.70	0.14	Rao et al., 2017^43^
Mortality after distant recurrence, 5-year	0.80	0.16	Rao et al., 2017^43^	0.80	0.16	Rao et al., 2017^43^	0.80	0.16	Rao et al., 2017^43^
Mortality after distant and local recurrence, 5-year	0.80	0.16	Rao et al., 2017^43^	0.80	0.16	Rao et al., 2017^43^	0.80	0.16	Rao et al., 2017^43^
Mortality, natural causes	2015 US Social Security Administration actuarial life tables			2015 US Social Security Administration actuarial life tables			2015 US Social Security Administration actuarial life tables		

*WW* watch and wait, *LAR* low anterior resection, *APR* abdominoperineal resection, *SD* standard deviation

### Base-Case Description

The standard (base-case) patient analyzed was a patient with locally advanced (T3, N Any or T1-2, N1-2) rectal cancer who achieved a complete clinical response following chemoradiation. To make the WW and LAR/APR groups comparable, we included only locally advanced (T3, N Any or T1-2, N1-2) disease. Therefore, all patients would undergo neoadjuvant chemoradiation. Based on National Comprehensive Cancer Network (NCCN) guidelines, all these patients were modeled to undergo adjuvant therapy to total 6 months’ duration of therapy. Given 1½ months of neoadjuvant chemoradiation, we modeled this as 4 months of adjuvant chemotherapy to start postoperatively, when patients entered the well state (2 months after resection). The base case for APR received permanent ostomies. LAR patients were modeled to have ostomies for 6 months postoperatively (4 months of chemotherapy + 2 months of recovery prior to ostomy reversal). All patients undergoing salvage for local recurrence/regrowth received APR or LAR, depending on the model.

In contrast to patients undergoing APR or LAR, patients monitored under WW protocols were modeled to directly enter the stable disease state for post-neoadjuvant treatment surveillance based on previously published Memorial Sloan-Kettering nonoperative management surveillance programs.^24^ WW patients could then remain with no evidence of disease or develop a potentially resectable local regrowth. Patients who underwent resection for local regrowth were then assigned to a postoperative stable disease state, in which they could then have a potentially salvageable local recurrence. We adopted this approach in modeling the post-neoadjuvant treatment course for WW patients to reflect the reality that WW does not preclude surgery, but rather offers patients an opportunity to avoid resection, reserving radical resection for local regrowth.

The model used a 1-month cycle length and extended over a 5-year time horizon. We used a 5-year time horizon because the majority (80%) of disease recurrences occur within 2 years of resection and/or treatment, with over 98% of recurrences falling within 5 years.^25^ Most of the available literature comparing WW versus radical resection is also limited to 5-year follow-up, thus limiting any attempt to expand our time horizon beyond 5 years without making assumptions or extrapolating data.^14^

We performed all model building and analysis using TreeAge Pro Healthcare version 2021 (Williamstown, MA, USA).

### Model Parameters

Probabilities of disease progression and cancer death for all arms in the study were determined from peer-reviewed sources (Table 1). Probability of death from natural causes was determined from Social Security life tables (Table 1). Effectiveness was measured in QALYs, which reflects the product of health utility and time. Health utility represents patient quality of life (measured in QALYs), which ranges from 0 (death) to 1 (perfect health). Each health state has a distinct health utility score, and the health utility after surgery depended on the presence or absence of an ostomy (Table 2).

**Table 2:** Transition and health state utility parameters (in QALYs) and their respective citations

Model parameter	WW			LAR			APR		
	Value	SD	Citation	Value	SD	Citation	Value	SD	Citation
*Utilities (β Distribution)*									
Initial state: WW versus surgery	0.80	0.16	Couwenberg et al., 2018^19^	0.110	0.022	van den Brink et al., 2004^20^	0.110	0.022	van den Brink et al., 2004^20^
Disutility of salvage TME	0.69	0.14	van den Brink et al., 2004^20^	0.690	0.14	van den Brink et al., 2004^20^	0.690	0.14	van den Brink et al., 2004^20^
Surgery, long-term	–	–	–	0.70, 0.78*	0.14, 0.16	Couwenberg et al., 2018^19^	0.750	0.15	Couwenberg et al., 2018^19^
Local recurrence	0.67	0.13	van den Brink et al., 2004^20^	0.670	0.13	van den Brink et al., 2004^20^	0.670	0.13	van den Brink et al., 2004^20^
Distant recurrence	0.70	0.14	van den Brink et al., 2004^20^	0.700	0.14	van den Brink et al., 2004^20^	0.700	0.14	van den Brink et al., 2004^20^
Distant and local recurrence	0.48	0.10	van den Brink et al., 2004^20^	0.480	0.096	van den Brink et al., 2004^20^	0.480	0.096	van den Brink et al., 2004^20^
Death	0.00	–	–	0.000	–	–	0.000	–	–

*WW* watch and wait, *LAR* low anterior resection, *APR* abdominoperineal resection, *SD* standard deviation, *TME* total mesorectal excision

Costs were determined from the payer perspective using Current Procedural Terminology (CPT) billing codes and the 2019 Physician’s Fee Schedule, and were adjusted to 2019 US dollars ($) using the Consumer Price Index to account for inflation.^26^ To more accurately reflect costs of cancer care, we incorporated time-dependent costs for the following components of treatment: chemotherapy, ostomy care, and post-treatment cancer surveillance costs. An exception to this was the cost of APR/LAR. This was because of the relative heterogeneity of CPT billing codes used to document such operations. Moreover, reliance on CPT billing codes does not reflect the varied experiences in anesthesia and inpatient admissions. To ensure a more standard reporting of operative cost, we used a previously published source instead of the Consumer Price Index.^27^ The same source was used to determine the costs of adjuvant chemotherapy. Surveillance after surgery was based on the American Society of Clinical Oncology guidelines^28^ (Table 3).

**Table 3:** Transition and health state cost parameters (in US$) and their respective citations

Model parameter	WW			LAR			APR		
	Value	SD	Citation	Value	SD	Citation	Value	SD	Citation
*Cost (gamma distribution)*^*b*^									
Surgery	Salvage TME costs reflect APR or LAR costs, depending on the model			$34,662.98	$6932.60	Raldow et al., 2019^27^	$22,015.72	$4403.14	Raldow et al., 2019^27^
Adjuvant chemotherapy, per 28-day cycle (also used for adjuvant chemotherapy following re-irradiation)	$1890.00	$378.00	Raldow et al., 2019^27^	$1890.00	$378.00	Raldow et al., 2019^27^	$1890.00	$378.00	Raldow et al., 2019^27^
Ostomy take-down	$906.02	$181.20	2019 Physician's Fee Schedule^27^	$906.02	$181.20	2019 Physician's Fee Schedule^27^	–	–	–
Ostomy care, 1-month	$93.25	$18.65	2020 DMEPOS Schedule^54^	$93.25	$18.65	2020 DMEPOS Schedule^54^	$93.25	$18.65	2020 DMEPOS Schedule^54^
Office visit	$25.95	$5.19	2019 Physician's Fee Schedule^26^	$25.95	$5.19	2019 Physician's Fee Schedule^26^	$25.95	$5.19	2019 Physician's Fee Schedule^26^
Rectal examination	$9.01	$1.80	2019 Physician's Fee Schedule^26^	$9.01	$1.80	2019 Physician's Fee Schedule^26^	–	–	–
Flexible sigmoidoscopy	$58.74	$11.75	2019 Physician's Fee Schedule^26^	$58.74	$11.75	2019 Physician's Fee Schedule^26^	–	–	–
CEA	$23.41	$4.68	2019 Physician's Fee Schedule^26^	$23.41	$4.68	2019 Physician's Fee Schedule^26^	$23.41	$4.68	2019 Physician's Fee Schedule^26^
Colonoscopy through stoma	$164.34	$32.87	2019 Physician's Fee Schedule^26^	–	–	–	$164.34	$32.87	2019 Physician's Fee Schedule^26^
Colonoscopy	$194.97	$38.99	2019 Physician's Fee Schedule^26^	$194.97	$38.99	2019 Physician's Fee Schedule^26^	$194.97	$38.99	2019 Physician's Fee Schedule^26^
CT abdomen, pelvis with contrast	$323.99	$64.80	2019 Physician's Fee Schedule^26^	$323.99	$64.80	2019 Physician's Fee Schedule^26^	$323.99	$64.80	2019 Physician's Fee Schedule^26^
CT chest, without contrast	$161.09	$32.22	2019 Physician's Fee Schedule^26^	$161.09	$32.22	2019 Physician's Fee Schedule^26^	$161.09	$32.22	2019 Physician's Fee Schedule^26^
MRI pelvis, with/without contrast	$405.08	$81.02	2019 Physician's Fee Schedule^26^	$405.08	$81.02	2019 Physician's Fee Schedule^26^	$405.08	$81.02	2019 Physician's Fee Schedule^26^
Clinical evaluation and restaging for recurrent disease:									
Local regrowth or pelvic recurrence	$2100.00	$420.00	Miller et al., 2020^41^	$2100.00	$420.00	Miller et al., 2020^41^	$2100.00	$420.00	Miller et al., 2020^41^
Distant recurrence	$1200.00	$240.00	Miller et al., 2020^41^	$1200.00	$240.00	Miller et al., 2020^41^	$1200.00	$240.00	Miller et al., 2020^41^
Re-irradiation for pelvic recurrence, single course	$19,800.00	$3960.00	Miller et al., 2020^41^	$19,800.00	$3960.00	Miller et al., 2020^41^	$19,800.00	$3960.00	Miller et al., 2020^41^
Chemotherapy for recurrent/metastatic disease:									
Palliative capecitabine for unsalvageable pelvic recurrence, per cycle	$510.00	$102.00	Miller et al., 2020^41^	$510.00	$102.00	Miller et al., 2020^41^	$510.00	$102.00	Miller et al., 2020^41^
Palliative mFOLFOX6 for distant metastasis, per cycle	$850.00	$170.00	Miller et al., 2020^41^	$850.00	$170.00	Miller et al., 2020^41^	$850.00	$170.00	Miller et al., 2020^41^
Perioperative death	$11,295.38	$2259.08	Duncan et al., 2019^55^	$11,295.38	$2259.08	Duncan et al., 2019^55^	$11,295.38	$2259.08	Duncan et al., 2019^55^
Cancer-related death	$11,295.38	$2259.08	Duncan et al., 2019^55^	$11,295.38	$2259.08	Duncan et al., 2019^55^	$11,295.38	$2259.08	Duncan et al., 2019^55^
Non-cancer death	$11,295.38	$2259.08	Duncan et al., 2019^55^	$11,295.38	$2259.08	Duncan et al., 2019^55^	$11,295.38	$2259.08	Duncan et al., 2019^55^

^a^ With, without ostomy, respectively

^b^ In 2019 US dollars

*WW* watch and wait, *LAR* low anterior resection, *APR* abdominoperineal resection, *SD* standard deviation, *TME* total mesorectal excision, *CEA* carcinoembryonic antigen, *CT* computed tomography, *MRI* magnetic resonance imaging, *DMEPOS* Durable Medical Equipment, Orthotics or Prosthetics

### Example of Transition Through Model

A base-case patient randomized to surgery may have stable disease for 1 year until they develop local recurrence not eligible for surgical resection. Within the model, this patient would cycle 12 times (one cycle/month) within the stable disease health state then progress to local recurrence without salvage. This patient may then develop distant recurrence 6 months later. Within the model, this patient would cycle within the local recurrence without salvage state for six cycles (i.e. months) before progressing to the local and distant recurrence stage. As the patient cycles through each health state, there is a chance of death from natural causes, a chance to remain in said health state, or a chance to transition to a different health state. Each transition and health state has an associated cost ($) and utility (QALYs) that accumulates as a patient progresses through the model until the time horizon or death is reached.

### Statistical Analysis

Cost effectiveness was assessed with an incremental cost-effectiveness ratio (ICER), which represents the incremental costs divided by incremental QALYs of each treatment group. ICERs under $100,000/QALY were considered cost effective. The willingness-to-pay (WTP) threshold is defined as the ICER below which an intervention is considered cost-effective. We used $100,000 as the threshold based on previously published literature as well as other peer-reviewed cost-effectiveness analyses.^29–31^ Treatments that lowered costs and increased effectiveness were considered dominant. All costs and utilities were discounted by 3% annually with half-cycle corrections. We conducted one-way (i.e. deterministic) sensitivity analysis on all probabilities, utilities, and costs to determine their impact on cost effectiveness. For transition probabilities and utilities in our one-way sensitivity analysis, we used the broadest range of values possible (i.e. as close to 0–100%), and for cost parameters, we used a lower and upper bound of 5% and 195% the base cost parameter, respectively. We conducted probabilistic sensitivity analysis where we modeled transition probabilities and health utilities with β distributions, and costs with γ distributions. We identified standard deviations for each variable distribution from the literature and used a standard deviation equal to 20% of the mean with unknown standard deviations. We tested different values of our unknown standard deviation (ranging from 10% to 40% of the mean) in a sensitivity analysis, which did not impact our results (analysis not shown). This study was conducted and published according to previously reported guidelines.^32^

### Human Subjects

We did not use data generated directly from human subjects in our work.

## Results

### Base-Case Microsimulation

In our APR versus WW base-case microsimulation, APR cases had a slightly higher 5-year overall mortality rate (14.7% vs. 13.7%) but lower 5-year cancer-specific mortality (4.7% vs. 6.1%) compared with WW cases. Similarly, in the LAR versus WW base-case microsimulation, LAR cases had higher 5-year overall mortality (14.8% versus 13.2%) with lower cancer-specific mortality (4.5% versus 6.0%) compared with WW. This is likely due to a perioperative death risk that both LAR and APR patients are exposed to in higher rates than WW patients (Table 1). In both APR versus WW and LAR versus WW base microsimulations, surgical resection had lower rates of distant and combined recurrences than WW cases. Although WW arms had higher rates of local regrowth prior to salvage than LAR/APR had after surgery, rates of local recurrence in the WW arms following salvage were comparable with APR and LAR (Table 4). Our model was validated by comparing these outcomes with values in published literature.

**Table 4:** Five-year survival and recurrence outcomes of the base-case analysis

	APR	WW	LAR	WW
Recurrence, 5-year				
Local regrowth (during the WW period)	–	31.25	–	30.83
Local recurrence (following first operation)	3.69	2.33	3.37	2.37
Distant only	7.52	10.32	7.40	10.37
Local and distant (includes concurrent local regrowth if WW)	1.46	2.93	1.33	3.07
Mortality, 5-year				
Overall	14.71	13.71	14.79	13.22
Cancer-specific	4.69	6.14	4.48	5.99

Data are expressed as percentages

*WW* watch and wait, *LAR* low anterior resection, *APR* abdominoperineal resection

Our microsimulation model of LAR versus WW found that LAR incurred $50,484.77 for 3.24 QALYs versus $26,499.86 and 3.41 total QALYs for WW. This led to WW decreasing costs by $23,984.91 and increasing effectiveness by 0.17 QALYs compared with LAR. In our APR versus WW microsimulation model, APR incurred $40,655.57 for 3.17 QALYs, while WW incurred $23,894.94 for 3.40 QALYs. This led to WW decreasing costs by $16,760.63 and increasing effectiveness by 0.23 QALYs compared with APR (Table 5). By reducing costs and increasing effectiveness, WW dominated both LAR and APR.

**Table 5:** Outcomes of the base-case analysis

	APR	WW	LAR	WW
Cost	$40,655.13	$23,894.94	$50,484.77	$26,499.86
Effectiveness (QALY)	3.17	3.40	3.24	3.41
Incremental cost (vs. WW)	$16,760.63		$23,984.91	
Incremental effectiveness (vs. WW)	− 0.23		− 0.17	
ICER ($/QALY)	Dominated		Dominated	

*WW* watch and wait, *LAR* low anterior resection, *APR* abdominoperineal resection, *QALY* quality-adjusted life-year, *ICER* incremental cost-effectiveness ratio

### Deterministic Sensitivity Analysis

We found that both microsimulations were sensitive to 2-year local regrowth rates following WW, 5-year distant recurrence rates following WW, and WW utility. In addition, our APR versus WW microsimulation was sensitive to APR utility, and our LAR versus WW microsimulation was sensitive to LAR utility following ostomy reversal (Table 6). Specifically, both APR and LAR became more cost-effective than WW if 2-year local recurrence rates following WW exceeded 86 and 83%, respectively. Similarly, APR and LAR were more cost-effective than WW when 5-year distant recurrence rates exceeded 57 and 56%, respectively. APR was more cost effective than WW when utility after APR was higher than for WW. Similarly, LAR was more cost effective when utility after LAR and ostomy reversal was higher than for WW.

**Table 6:** Results of the deterministic sensitivity analysis

Model parameter	Base value	Range of values		APR relative to WW			LAR relative to WW		
		Lower bound	Upper bound	WW dominates	APR not cost effective	APR cost effective	WW dominates	LAR not cost effective	LAR cost effective
*Probabilities (β distribution)*									
Perioperative death	0.035	0.001	0.9	Remains dominated			Remains dominated		
Local recurrence, 2 years, WW	0.19	0.16	1	<0.82	0.82–0.86	>0.86	<0.66	0.66–0.83	>0.83
Local recurrence, 5 years, WW	0.24	0.2	1	Remains dominated			<0.99998877	0.99998877-1	1
Local recurrence, 2 years, TME	0.016	0.01	1	Remains dominated			Remains dominated		
Local recurrence, 5 years, TME	0.01	0.005	1	Remains dominated			Remains dominated		
Salvage for local recurrence, WW	0.941	0.1	0.95	Remains dominated			Remains dominated		
Salvage for local recurrence, TME	0.63	0.01	0.8	Remains dominated			Remains dominated		
Distant recurrence, 5-year, WW	0.098	0.05	1	<0.39	0.39–0.57	>0.57	<0.47	0.47–0.56	>0.56
Distant recurrence, 5-year, TME	0.079	0.05	1	Remains dominated			Remains dominated		
Concurrent diagnosis of distant recurrence if local recurrence, WW	0.056	0.01	0.9	<0.78	>0.78	–	<0.57	>0.57	–
Concurrent diagnosis of distant recurrence if local recurrence, TME	0.056	0.01	0.9	Remains dominated			Remains dominated		
Concurrent diagnosis of local recurrence if distant recurrence, WW	0.169	0.01	0.9	Remains dominated			Remains dominated		
Concurrent diagnosis of local recurrence if distant recurrence, TME	0.169	0.01	0.9	Remains dominated			Remains dominated		
Distant recurrence following local recurrence, 3-year, WW	0.113	0.01	1	Remains dominated			Remains dominated		
Distant recurrence following local recurrence, 3-year, TME	0.113	0.01	1	Remains dominated			Remains dominated		
Mortality after local recurrence with salvage, 5-year	0.5	0.1	1	Remains dominated			Remains dominated		
Mortality after local recurrence without salvage, 5-year	0.7	0.1	1	Remains dominated			Remains dominated		
Mortality after distant recurrence, 5-year	0.8	0.1	1	Remains dominated			Remains dominated		
Mortality after distant and local recurrence, 5-year	0.8	0.1	1	Remains dominated			Remains dominated		
Mortality, natural causes, monthly	US SSA life tables	0.001	0.9	<0.18	>0.18	–	<0.31	>0.31	–
*Utilities (β distribution)*									
Initial TME	0.11	0.05	0.95	Remains dominated			Remains dominated		
WW, long-term	0.8	0.05	0.95	0.76 and above	0.68–76	0.05–0.68	>0.74	0.68–0.74	0.05–0.68
Disutility of salvage TME	0.69	0.05	0.95	Remains dominated			0.05–0.81	>0.81	–
Surgery, long-term									
APR	0.75	0.1	0.9	0.1–0.86	0.86–0.88	0.88–0.9	–		
LAR with ostomy	0.7	0.05	0.95	–			Remains dominated		
LAR without ostomy	0.78	0.05	0.95	–			0.05–0.84	0.84–0.93	>0.93
Local recurrence	0.67	0.01	0.95	Remains dominated			Remains dominated		
Distant recurrence	0.7	0.01	0.95	Remains dominated			Remains dominated		
Distant and local recurrence	0.48	0.0048	0.96	Remains dominated			Remains dominated		
Death	0	–	–	–	–	–	–	–	–
*Cost (γ distribution)*^*a*^									
Surgery									
LAR	$34,662.98	$1733.15	$67,592.81	–	–	–	Remains dominated		
APR	$22,015.72	$1100.79	$42,930.65	Remains dominated			–	–	–
Adjuvant chemotherapy, per 28-day cycle (also used for adjuvant chemotherapy following reirradiation)	$1890	$94.50	$3685.50	Remains dominated			Remains dominated		
Ostomy take-down	$906.02	$45.30	$1766.74	Remains dominated			Remains dominated		
Ostomy care, 1-month	$93.25	$4.66	$181.84	Remains dominated			Remains dominated		
Office visit	$25.95	$1.30	$50.60	Remains dominated			Remains dominated		
Rectal examination	$9.01	$0.45	$17.57	Remains dominated			Remains dominated		
Flexible sigmoidoscopy	$58.74	$2.94	$114.54	Remains dominated			Remains dominated		
CEA	$23.41	$1.17	$45.65	Remains dominated			Remains dominated		
Colonoscopy through stoma	$164.34	$8.22	$320.46	Remains dominated			Remains dominated		
Colonoscopy	$194.97	$9.75	$380.19	Remains dominated			Remains dominated		
CT abdomen, pelvis with contrast	$323.99	$16.20	$631.78	Remains dominated			Remains dominated		
CT chest, without contrast	$161.09	$8.05	$314.13	Remains dominated			Remains dominated		
MRI pelvis, with/without contrast	$405.08	$20.25	$789.91	Remains dominated			Remains dominated		
Clinical evaluation and restaging for recurrent disease									
Local regrowth or pelvic recurrence	$2100.00	$105	$4095	Remains dominated			Remains dominated		
Distant recurrence	$1200.00	$60	$2340	Remains dominated			Remains dominated		
Reirradiation for pelvic recurrence, single course	$19,800.00	$990	$38,610	Remains dominated			Remains dominated		
Chemotherapy for recurrent/metastatic disease									
Palliative capecitabine for unsalvageable pelvic recurrence, per cycle	$510.00	$25.50	$994.50	Remains dominated			Remains dominated		
Palliative mFOLFOX6 for distant metastasis, per cycle	$850.00	$42.50	$1657.50	Remains dominated			Remains dominated		
Perioperative death	$11,295.38	$564.77	$22,025.99	Remains dominated			Remains dominated		
Cancer-related death	$11,295.38	$564.77	$22,025.99	Remains dominated			Remains dominated		
Non-cancer death	$11,295.38	$564.77	$22,025.99	Remains dominated			Remains dominated		

*WW* watch and wait, *LAR* low anterior resection, *APR* abdominoperineal resection, *TME* total mesorectal excision, *SSA* Social Security Administration, *CEA* carcinoembryonic antigen, *CT* computed tomography, *MRI* magnetic resonance imaging

^a^ In 2019 US dollars

Despite some evidence suggesting that delaying adjuvant treatment past 4 weeks was detrimental to outcomes, timing of chemotherapy initiation ranged between 4 and 12 weeks among published randomized trials.^33–38^ We thus performed a sensitivity analysis of this parameter. For each tree, we modeled adjuvant chemotherapy to start at 4, 8, and 12 weeks post-intervention. In all scenarios, WW remained dominant over LAR and APR.

### Probabilistic Sensitivity Analysis

Our probabilistic sensitivity analysis (100 microsimulations of 10,000 cases) for both LAR and APR models versus WW found that WW was dominant or was considered cost effective over both strategies across a range of WTP thresholds from $0–$200,000/QALY in 100% of the iterations tested.

## Discussion

We used two separate Markov models, one comparing APR with WW, and one comparing LAR with WW, to simulate 10,000 cases per model. At a WTP threshold of $100,000, WW was the dominant strategy over both LAR and APR. In fact, even when increasing WTP thresholds to $250,000, the WW strategy remained dominant. Thus, WW was significantly more cost effective than resection.

Both APR and LAR models indicated that surgery was associated with increased 5-year mortality but decreased cancer-specific mortality. Overall, the findings suggest that patients undergoing surgery, regardless of type, seem to pay a mortality penalty (unrelated to cancer) that is greater in magnitude than the increased cancer-specific mortality associated with WW. These treatment-based differences can logically be attributed to both the risks associated with surgery and the marginal increased risk of leaving residual cancer in place with the WW strategy.

Based on these data, we suggest considering the WW strategy for patients with cCR; however, we must also acknowledge the effects on distant recurrence. Both distant metastases, and combined local and distant metastases, were slightly more common in patients treated with WW. This observation, demonstrating increased metastatic potential when the primary tumor is left in situ, is consistent with a priming role for the primary tumor, where the primary tumor sends signals to distant sites to prepare them for metastatic seeding.^39^ However, this supposes remnant tumor tissue and it is unclear whether tissue recently cleared of malignancy might assume a similar role.

While these numbers are interesting to examine, they are principally meant to confirm that the model functions as expected. Thus, we must also determine whether our findings might be affected by factors built into the model. Both models were sensitive to regrowth rates, distant recurrence, and utilities associated with WW or following surgery. In both models, cost effectiveness of WW was sensitive to rates of regrowth and distant recurrence after WW. From a clinical standpoint, most hesitation to embrace WW stems from the possibility that the strategy may put patients at higher risk of developing unresectable or distant metastatic disease. In the APR versus WW model, the 2-year local regrowth rate during WW would have to be >86% to make APR more cost effective than WW. Similarly, a threshold above 90% 2-year local regrowth after WW would result in LAR being more cost effective. Both thresholds are much higher than the maximum 30% regrowth reported in the literature.^14,16,40^ The extreme nature of these thresholds may be due to the high salvage rates that we modeled, taken from the current literature. We also found that surgery (either LAR or APR) was only more cost effective if rates of 5-year distant metastatic disease exceeded 57% in the WW group. Again, this theoretical threshold is much higher than accepted distant recurrence rates of up to 13%.^14^ Therefore, from a cost-effectiveness perspective, WW is superior to both LAR and APR when clinically relevant regrowth and distant metastatic recurrence rates are considered.

Of note, our models were not sensitive to cost. Although various surveillance schedules after WW have been cited in literature, we did not vary the surveillance schedule in our model, which was based on just one of several published WW protocols.^28^ Altering the surveillance schedule might affect this result; however, we expect these results are robust and would remain unaltered since the model was not sensitive to cost in any capacity. Additionally, other groups who have approached modeling the data with various strategies, have arrived at a similar conclusion regarding cost.^41^

Theoretically, APR and LAR could be more cost effective than WW if patient-reported quality of life following radical surgery was better than after WW^19^ (Table 6). However, these criteria are clinically unrealistic; we would rarely expect a patient who has had only chemoradiation to report worse quality of life than a patient who has had chemoradiation followed by radical surgery and possibly a permanent stoma. This is supported by literature gathered regarding patient-reported quality of life, which have consistently ranked perceived quality of life to be higher following WW than either APR or LAR.^19,20^

While recurrence rates are a priority among providers, patient-centered outcomes, such as QALYs, are crucial to acknowledge as well. Our model showed that implementing WW for rectal cancer patients with cCR after neoadjuvant therapy did not lead to an appreciable overall survival trade-off compared with either LAR or APR. However, WW had higher rates of local regrowth and marginally increased distant recurrence than both LAR and APR (Table 4). Despite this, WW offered a QALY and cost benefit compared with either operative approach. This is likely because cost-effectiveness analyses utilize a holistic approach balancing clinical outcomes, patient perspectives, and costs in a way that most studies focused on a single facet of patient care cannot offer.

Because WW dominated both LAR and APR in our base-case analyses, we did not expect WTP thresholds to significantly change our results. However, given the inherently subjective nature of WTP thresholds, we performed a probabilistic sensitivity analysis to test our model against a wide range of WTP thresholds ($0–$200,000/QALY), which showed that both LAR and APR remained dominated by WW independent of the WTP threshold.

Few cost-effectiveness analyses on WW versus radical surgery have been published in the past decade. Neuman et al. conducted the first analyses in 2009 and their model found surgery to be cost effective compared with WW.^42^ While informative, their model was built on older publications, including studies with relatively small patient numbers and using expert opinion for critical values such as recurrence rates and health utilities. In contrast, Rao et al. and Smith et al. published a similar analysis in 2015 and 2017, respectively, that found WW to be cost effective compared with radical surgery;^43,44^ however, these results were specific to elderly patients and, once more, used numbers based on expert opinion alone. Moreover, neither study stratified analysis by type of operation (i.e. LAR versus APR), which has clear clinical effects on postoperative morbidity, mortality, and quality of life.^45,46^

The most recently published cost-effectiveness analysis by Miller et al. also concluded that WW was the dominant strategy compared with radical surgery.^41^ One difference between our models is our perioperative death parameter. While Miller et al. cite a perioperative mortality rate of 0.6%, we modeled a rate of 3.5%. Our parameter value is derived from a prospective multicenter randomized trial studying locally advanced rectal cancer patients in whom neoadjuvant therapy was an inclusion criterion.^47^ In contrast, Miller et al. used a more recent retrospective study that included all rectal cancer patients whether or not they received neoadjuvant treatment.^48^ Furthermore, while the newer ROLARR trial may have provided lower perioperative mortality risks, less than half of their cohorts received neoadjuvant treatment.^49^ Nonetheless, a sensitivity analysis, accounting for extreme ranges in perioperative mortality (from 0.1% to 90%), showed that the perioperative mortality rate did not impact our results, therefore we maintained our analysis using mortality according to Marijnen et al. (Table 6).^47^

To complement and expand upon these models, we built two different models to capture outcomes unique to APR and LAR. In comparison, the model employed by Miller et al. permitted all patients the option of undergoing WW, LAR, or APR. However, APR is primarily reserved for rectal tumors that cannot be resected with an adequate margin using a sphincter-sparing approach, therefore patients who can undergo LAR and those who require an APR may have inherently different, preoperatively determined disease profiles, which might affect the cost effectiveness.^50^ As such, we believed two separate models were necessary. We also used a microsimulation, which, as discussed previously, allowed us to mimic the progress of individual patients. In addition, we allowed WW patients the opportunity to undergo two salvage surgeries (allowing for initial resection after regrowth as well as resection after recurrence if that occurred) as opposed to just one surgical intervention in the study by Miller et al. Our model also built-in greater granularity for regrowth/recurrence by allowing patients to experience both local regrowth and distant recurrence with different probabilities if distant recurrence was diagnosed first, and vice versa.^16^

These added intricacies can be attributed to our interdisciplinary approach to building and validating the model. Other differences include variations in sources of probabilities and utilities; we used the highest level of evidence from recently published data in well-established journals.^14–16,19^ All model utilities were drawn from an updated prospective survey by the PLCRC, which included more granular results.^19^ In contrast, prior cost-benefit analyses relied on less precise, prospective utility surveys, or on utilities for other disease processes such as prostate cancer.^43^

Limitations to this study are inherent to any cost-effectiveness analysis. Intrinsic to their nature, models simplify complex diseases and processes involving outcomes, patient perspectives, access to care, and other social factors. For example, we were also unable to evaluate the impact of patient compliance on surveillance and treatment due to the lack of robust studies with details on real-world follow-up. These effects may be especially important within the WW protocol given the need for regular follow-up, although some data suggest that differences in compliance may not affect patient outcomes.^17^ Still, since we used real-world data from retrospective studies to build our models, impacts of patient compliance may be inherent in the transition probabilities we used. Other studies have also suggested that adherence to post-operative chemotherapy is lower than expected.^51^ In contrast, our model assumed that all patients undergoing surgery also underwent adjuvant chemotherapy. Our goal was to have the model align as closely as possible to the standard of care and was also built upon the precedence set by prior decision models on a similar topic.^43^ Despite this, our assumption of perfect adherence to adjuvant chemotherapy is made across all interventions in our models: LAR, APR, and WW, thus controlling for any potential confounding that treatment adherence may confer.

Our reliance on costs from a payor perspective may appear to discount the very real costs experienced by patients. However, we stress that our health utilities, as measured in QALYs as outlined in our Methods section, are a useful surrogate for measuring the impacts (financial or otherwise) that diseases and their treatments have on our patients. Indeed, one can argue that financial impacts have real consequences on a patient’s perceived quality of life—a metric that is captured by the QALYs documented in the current literature.^19^ Moreover, given the heterogenous nature of healthcare reimbursement in the US, costs from the payor (i.e. Medicare) perspective are often the best available standard measure.^52^

Additionally, models are only as accurate as the studies used to build them. The decision to pursue surgery will have long-term ramifications on patient survival and quality of life, but our model ended at 5 years due to the lack of robust follow-up data beyond then. Despite these limitations, we included the most granular and high-level data available, and our model was validated by an interdisciplinary team all regularly involved in the care for this patient population.

Lastly, this model is not intended to predict treatment outcomes (i.e. recurrence rates or mortality) for this patient population. Our goal is to understand how treatment options for this specific patient population differ from a cost and quality-of-life perspective. As such, external validation is not standard for cost-effectiveness modeling. Nonetheless, we found that our model outcomes for recurrence rates and mortality were comparable with those in the current literature. Ultimately, a randomized clinical trial will be needed to verify the clinical outcomes of each treatment approach. One such trial, NORWAIT, is currently underway in Norway (NCT03402477).

## Conclusions

The results of our analyses suggest WW confers superior QALYs at lower costs when compared with radical surgery for patients with cCR after neoadjuvant chemoradiation for locally advanced rectal cancer. Based on our model, these benefits do not come at the expense of reduced overall survival. The WW strategy requires attention to detail in patient selection and unyielding diligence in surveillance. However, for eligible patients, it is a cost-saving approach that offers significantly improved quality of life without compromising oncologic outcomes.

## Dislcosure

Christina Liu Cui, William Yu Luo, Bard Clifford Cosman, Daniel Simpson, and Sonia Ramamoorthy have no disclosures to declare.
